# A retrospective comparative case series of efficacy and safety of radiofrequency thermocoagulation versus drug therapy in patients with trigeminal neuralgia: A clinical case report

**DOI:** 10.1097/MD.0000000000039353

**Published:** 2024-09-20

**Authors:** Jing Fu, Zhiqiang Nie, Yanfeng Zhang, Min Tang, Jianguo Guo

**Affiliations:** a Department of Stomatology, Hangzhou Xixi Hospital, Hangzhou, China; b School of Pharmacy, Guizhou Medical University, Gui’an New District, China; c Department of Pain, The First Affiliated Hospital, Zhejiang University School of Medicine, Hangzhou, China; d Department of Neurology, The First Affiliated Hospital, Zhejiang University School of Medicine, Hangzhou, China

**Keywords:** carbamazepine, radiofrequency thermocoagulation, trigeminal neuralgia

## Abstract

**Background:**

The trigeminal neuralgia (TN) is characterized by a unilateral, episodic, electric shock-like pain in the distribution of the trigeminal nerve. Both drug therapy and radiofrequency thermocoagulation (RT) are used to treat TN.

**Objective:**

To compare the efficacy and safety of RT and drug therapy in patients with TN.

**Methods:**

Between October 2020 and December 2022, 62 patients with TN were allocated to undergo TN treatment (group A) or drug therapy (group B). In group A, 30 patients received RT treatment, whereas 32 patients in group B receive drug treatment. Pain relief, clinical outcomes, and adverse events in both groups were evaluated.

**Results:**

And significantly greater reduction in Visual Analogue Scale scores was noted in group A than in group B in the initial 2-week period (*P* < .05). The excellent rate was 93.3% (28/30) in group A, whereas it was 68.8% (22/32) in group B during the initial 2-week period (*P* < .05). A total of 62 patients were followed up at least 12 months, with a mean follow-up time of 14.5 months. But there were no statistically significant differences between the 2 groups at the final follow-up. A total of 24 patients had facial numbness in group A. In contrast, ten patients in group B complained of discomfort including sedation, dizziness, nausea, vomiting. During the follow-up period, 4 patients in group A and 6 patients in group B experienced recurrent pain.

**Conclusion:**

RT is a safety and effective treatment for patients with classic TN, providing more benefits of quicker pain relief and higher patient’s satisfaction, compared with traditional drug therapy.

## 1. Introduction

The trigeminal neuralgia (TN) is characterized by a unilateral, episodic, electric shock-like pain in the distribution of the trigeminal nerve.^[1–3]^ A number of treatment options have been developed to relieve pain for patients with TN, including drug therapy, radiofrequency thermocoagulation (RT), stereotactic radiosurgery, balloon compression and microvascular decompression.^[1–3]^ Drug therapy remains the first-line treatment option and is usually effective in most cases, while other techniques are considered an alternative therapy for patients with TN unresponsive to conservative treatment.

The RT technique was initially developed by Réthi in 1913, and subsequently the modified procedure has been used as a minimally invasive treatment of TN in the 1970s.^[4]^ Previous studies demonstrated the RT technique can provide effective pain relief immediately after surgery.^[5–7]^ However, RT also has disadvantages and complications compared with drug therapy. Repeated puncture and improper position of needle may bring unnecessary damage to surrounding tissue. Pain recurrence and facial numbness were also detected during the follow-up time.^[4–7]^ To date, few clinical studies available directly compare the outcomes of RT and drug therapy in the treatment of TN. In the present study, we prospectively evaluate the efficacy and safety of these 2 treatments, with purpose of providing a basis for clinicians to make informed choices.

## 2. Patients and Methods

**Figure 1.**
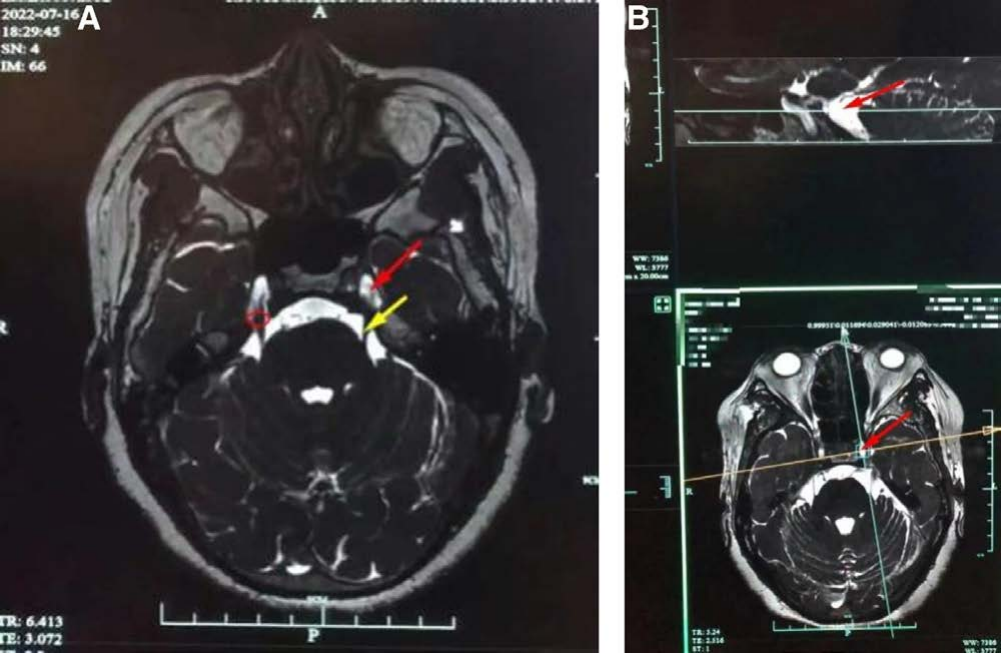
MRI scans of the left trigeminal nerve. Red arrow: the trigeminal ganglion; Yellow arrow: the trigeminal nerve; Red circle: the trigeminal nerve pore.

Between October 2020 and December 2022, a total of 62 patients with classic TN were enrolled in the study. The classic TN was diagnosed according to the International Headache Society criteria,^[8]^ including paroxysmal attacks, lasting from 1 second to 2 minutes, affecting 1 or more divisions of the trigeminal nerve; pain that is intense, sharp, superficial, or stabbing, precipitated from trigged areas; attacks stereotyped in the individual patient; absence of clinically evident neurological deficit; and pain not attributed to another disorder. Additionally, MRI is administered preoperatively for providing more diagnostic information of classic TN (Fig. 1).

This study was approved by our institutional committee for clinical research, and informed consent was obtained from the patients participating in the study. Patients were categorized into 2 groups based on the treatment they received as determined by the referring physician’s assessment and the patient’s preference. Group A consisted of patients who underwent radiofrequency thermocoagulation (RT), and Group B consisted of patients who received drug therapy. Secondary TN was excluded in all patients on magnetic resonance examination. Finally, the study population consisted of 30 patients in group A and 32 patients in group B. The demographic data of the patients in both groups are listed in Table 1. The 2 groups were similar with regard to patient age, gender, side, and pain region.

**Table 1:** Demographic data of the patients in both groups.

Variables		Group A (n = 30)	Group B (n = 32)	*P* value
Age		67.32 ± 10.52	66.47 ± 11.24	.336
Gender	Male	12	15	.585
	Female	18	17	
Side	Right	12	16	.429
	Left	18	16	
Pain distribution	V3	16	18	.818
	V2 + V3	14	14	

### 2.1. Interventions

**Figure 2.**
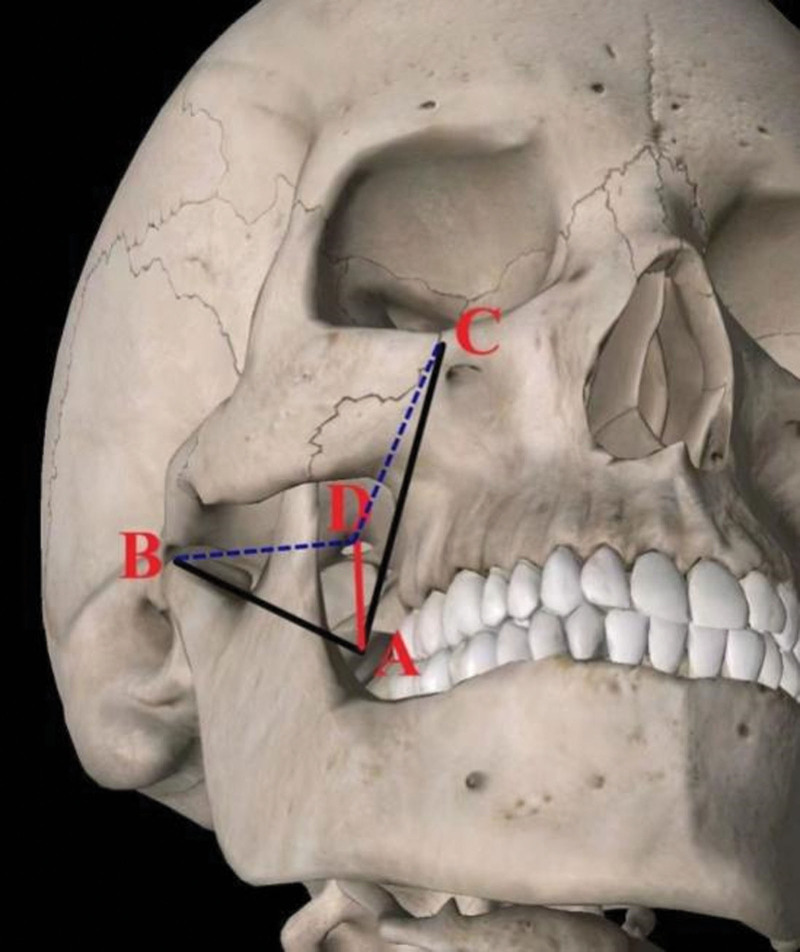
Schematic diagram of puncture.

**Figure 3.**
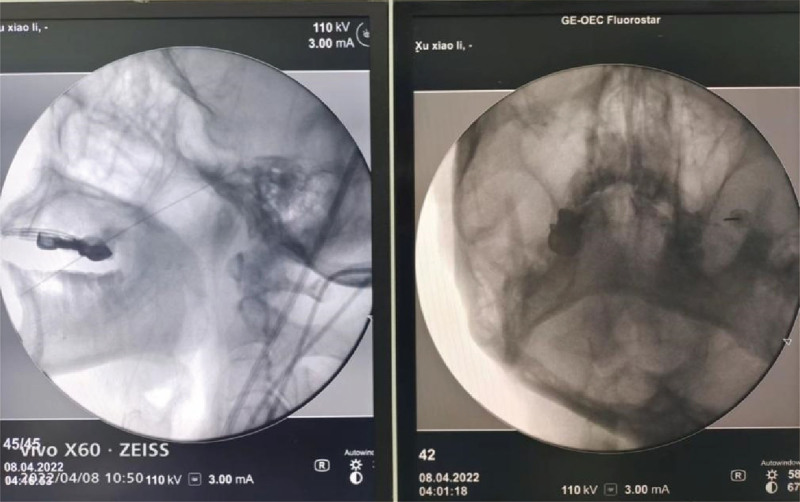
Intraoperative fluoroscopy images.

In group A, the patient lied in the supine position, and the head and neck were in a normal position. Generally, Hartel markings were drawn over the face (Fig. 2), including point A (the surgical puncture site, 3 cm outside the corner of the mouth), point B (the central lower part of the pupil), the front 2 to 3 cm of the external auditory canal was point C (the central lower part of the pupil). The lines AB and AC were drawn on the patient’s face, with AB connecting points A and B, and AC connecting points A and C. The puncture needle was positioned such that it was perpendicular to both lines AB and AC, ensuring accurate targeting of the foramen ovale. Finally, the intersecting line between the 2 faces was set as AD, determining the direction of the puncture. A solution of 10% sterile povidone–iodine was used for topical sterilization. The puncture needle entered from point A to the depth of 5 to 6 cm along the direction of AD under local anesthesia. When the tip of needle was found in front of the foramen ovale under fluoroscopy, continued to slide along the bone surface until the needle tip slide into the foramen ovale (Fig. 3). The final depth of the puncture needle into the foramen ovale was determined according to the patient’s pain site and the anatomical features of the semilunar ganglion. The II and III branches could be blocked when the puncture needle entered 3mm into foramen ovale. And the block region could be extended to I branch when the tip of needle entered 5mm into foramen ovale. However, the depth of entry into the foramen ovale should not exceed 1 cm.

After the puncture procedure was completed, the test electrode was placed for testing. Motor stimulation was performed using 2 Hz with 0.1 to 1.0 V in order to observe the masseter muscle contraction. Keeping verbal contact with patient sensory stimulation was given at 50 Hz, from 0.1 to 0.5 V to evoke a tingling sensation in the affected region. Radiofrequency therapy instrument (2000B, Beiqi Company, China) was used for treatment. RF lesioning was performed at 70°C for the first procedure in patient’s life and at 72°C for the following ones; the time of the lesion was set in 60 seconds. If more than 1 branch was affected, another lesion was performed by repositioning the needle. After each repositioning, the stimulation test was repeated to search for paresthesia at the desired area. The sensitivity of the face and cornea were tested.

In group B, carbamazepine was prescribed for treatment of TN, with the starting dose of 0.2 g. The dose is then titrated by adding 100 mg every other day until an adequate response is achieved or intolerable side effects are encountered.

### 2.2. Outcome measurement

Clinical data were collected by 2 independent observers. Pain was quantified on a Visual Analogue Scale (VAS) of 0 = no pain to 10 = severe pain before treatment, 2 weeks after treatment and the final follow-up. Furthermore, at the initially 2 weeks, clinical outcomes were divided into 3 categories according to Liu criteria^[9]^: excellent, good, and poor. “Excellent” was used for those patients who were pain free; “good” used for those who were in pain but with VAS score ≦ 2 without medications at 48 hours; “poor” used for those who were in pain, with VAS score ≧ 3 with or without medications.

### 2.3. Adverse events

Recurrence was defined as a return of trigeminal pain, which had the same characteristics as preoperatively, with VAS score ≧ 3, and pain frequency ≧ 3 times a day without medication use. Any complication related to RT was recorded. The side effects encountered with drug therapy were also recorded during the follow-up time.

## 3. Results

**Figure 4.**
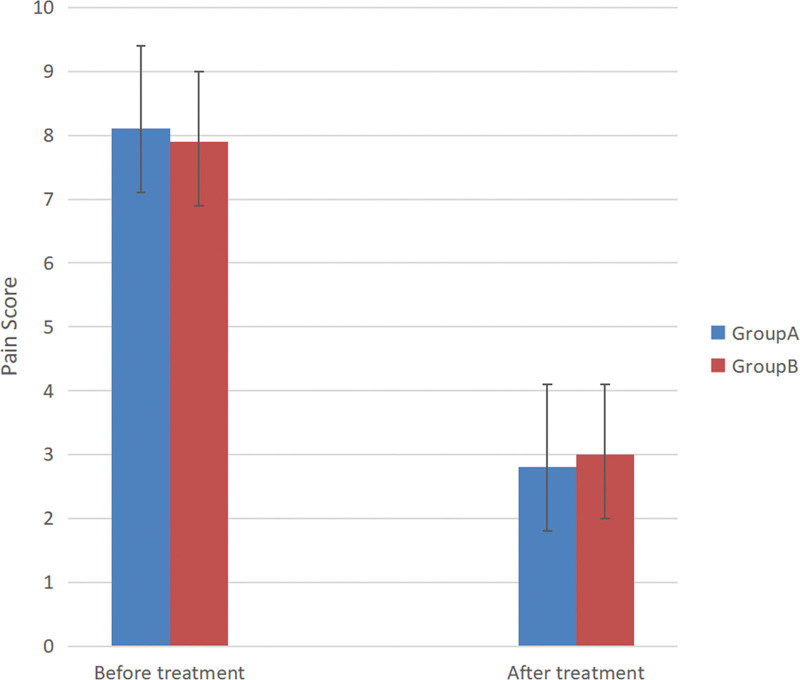
The graph of VAS pain score in both groups before and after treatment.

**Figure 5.**
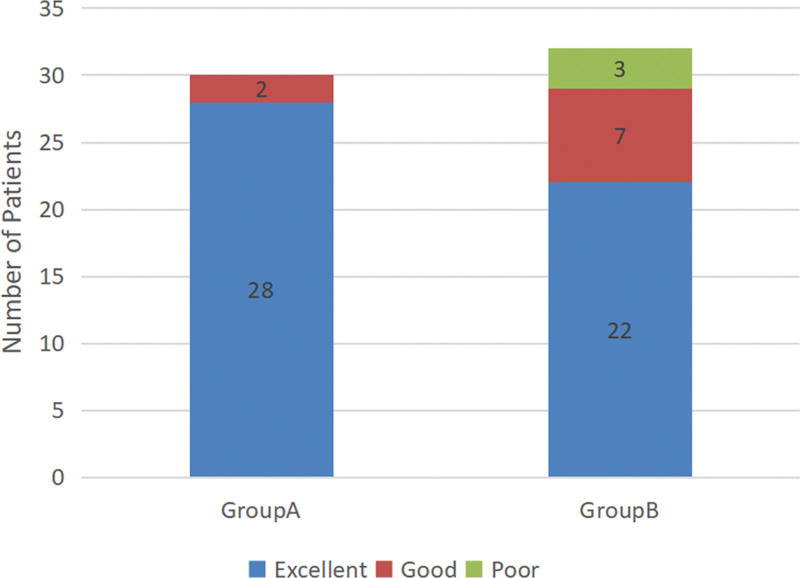
The status of pain relief at the initially 2 weeks in both groups.

The graph of VAS pain score was shown in Figure 4. Thirty patients in group A had significantly less pain after the RT procedures compared with their preoperative scores. The mean pain score decreased from 8.1 ± 1.6 before surgery to 2.8 ± 1.3 at 2 weeks after TN (*P* < .001). However, in group B, only 22 patients had remarkable pain alleviation. The mean pain score slight decreased from 7.9 ± 1.8 to 3.0 ± 1.1 at 2 weeks after drug therapy (*P* < .001). And significantly greater reduction in VAS scores was noted in group A than in group B in the initially 2 weeks period (*P* < .05). The status of pain relief at the initially 2 weeks was shown in Figure 5. The excellent rate was 93.3% (28/30) in group A, whereas it was 68.8% (22/32) in group B (*P* < .05). A total of 62 patients were followed up at least 12 months, with a mean follow-up time of 14.5 months. But there were no statistically significant differences were observed when mean pain scores were compared between the 2 groups at the final follow-up.

A total of 24 patients had facial numbness in group A, but disappeared within 6 months after surgery. Ten patients in group B complained of discomfort including sedation, dizziness, nausea, vomiting. Thus, the dosage of drug had to be adjusted until these symptoms were alleviated. Four patients who were refractory to drug therapy within the 2 weeks required TN treatment.

During the follow-up period, 4 patients in group A and 6 patients in group B experienced recurrent pain, respectively. Only 1 patient in group A and 3 in the group B required surgical interventions again. There was no significant difference in adverse response between the 2 groups (*P* > .05).

## 4. Discussion

TN is a sudden, unilateral, stabbing pain in the distribution of 1 or more branches of the trigeminal nerve. Although the exact mechanism of TN remains unknown, it is currently accepted that the etiology of TN is vascular compression of the central axons of the trigeminal nerve at the level of the pons, which results in focal demyelination.^[1,2]^

In China, TN is usually treated by neurologists, dentists and pain therapists. Despite treated in different departments, the sodium channel blockers remain the first choice drugs for conservative treatment of classic TN. Carbamazepine has been studied in several clinical trials since the 1960s and considered as the most effective drug.^[3,10]^ In the present study, 68.8% of the patients treated by carbamazepine obtain markedly pain relief during the 2 weeks period. These results were comparable to previous published reports.^[11,12]^ However, we noted that our patient’s satisfaction of the carbamazepine treatment is hampered by several side effects, including sedation, dizziness, nausea, vomiting. And 4 patients do not respond to this type of drug therapy, with the need for surgical treatment.

The RT procedure may be preferred for patients who are refractory to drug therapy. It is a minimally invasive ablative intervention through damage to the trigeminal axons in the nerve root or ganglion.^[4]^ In previous studies, RT therapy has demonstrated favorable clinical outcomes in the treatment of TN, with pain relief ranging from 77.8% to 100%.^[13]^ In the present study, 93.3% of patients obtained significantly pain control after RT treatment. Despite there were no statistically significant differences in mean VAS scores at the final follow-up, the benefits of RT procedure compared to drug therapy have been noted in our series, including quicker pain relief and patient satisfaction.

In the RT treatment, proper puncture procedures and accurate intraoperative position of the needle tip are essential for the success, while reducing the occurrence of complications and recurrence. In order to achieve these goals, we thought there are several key points in the RT procedure. Firstly, 3-dimensional imaging reconstruction of the skull base should be obtained prior to the surgery, which helps to find the anatomical variations and to avoid inadvertent injury to the surrounding neurovascular structures. Secondly, the patient position should be kept stable during the operation. Lastly but very importantly, the location into the foramen ovale and depth of penetration of the needle should be guided under fluroscopy, and the needle should be directed at the anterolateral aspect of the foramen ovale and slided into the foramen. In our series, no patients underwent anesthesia dolorosa, masseter weakness and dysesthesia after surgery. But 24 patients had facial numbness, which spontaneously resolved within 6 months postoperatively. Our results indicated that the standardized RT procedure is a safety treatment of classic TN.

Recurrence of pain is another important issue in the treatment for TN. In the present study, the recurrence rate for patients receiving RT was comparable to patients receiving drug therapy in the mean follow-up period of 14.5 months. Although the short-term prognosis is favorable in our series, long-term follow-up studies should be conducted to further examine recurrence rates of pain.

In conclusion, RT is a safe and effective treatment for patients with classic TN, providing more benefits of quicker pain relief and higher patient’s satisfaction, compared with traditional drug therapy.

## Acknowledgments

We would like to express our gratitude to the patients for their great help in this report.

## Author contributions

**Conceptualization:** Jing Fu, Zhiqiang Nie, Yanfeng Zhang, Min Tang, Jianguo Guo.

**Data curation:** Jing Fu, Zhiqiang Nie, Yanfeng Zhang, Jianguo Guo.

**Formal analysis:** Jing Fu, Zhiqiang Nie, Jianguo Guo.

**Methodology:** Yanfeng Zhang.

**Project administration:** Jianguo Guo.

**Supervision:** Jing Fu, Zhiqiang Nie, Jianguo Guo.

**Writing– original draft:** Jing Fu, Zhiqiang Nie, Jianguo Guo.

**Writing– review & editing:** Jing Fu, Zhiqiang Nie, Yanfeng Zhang, Min Tang, Jianguo Guo.
